# Inhibition of DHODH Enhances Replication-Associated Genomic Instability and Promotes Sensitivity in Endometrial Cancer

**DOI:** 10.3390/cancers15245727

**Published:** 2023-12-06

**Authors:** Shengyuan Zhao, Aaliyah Francois, Dawit Kidane

**Affiliations:** 1 Division of Pharmacology and Toxicology, Dell Pediatric Research Institute, College of Pharmacy, The University of Texas at Austin, 1400 Barbara Jordan Blvd. R1800, Austin, TX 78723, USA; 2 Department of Physiology and Biophysics, College of Medicine, Howard University, 520 W Street N.W., Washington, DC 20059, USA

**Keywords:** de novo nucleotide biosynthesis, DNA repair, DNA damage, endometrial cancer

## Abstract

**Simple Summary:**

Endometrial cancer is the most common type of cancer that affects the female reproductive organs. Over 50% of women with EC present with early-stage, low-risk disease, and are treated with surgery alone. However, women diagnosed with advanced or recurrent disease have a poor prognosis and they have poor response to current therapies. However, the molecular mechanisms and the prognostic prediction for EC patients remain unclear. In this work, we identified that cancer cells utilize dysregulated nucleotide metabolism to enhance proliferation and progression. We targeted one of the dysregulated genes (DHODH) that is critical for metabolism of nucleotide biosynthesis in endometrial cancer cell. Our results provide potential clinical application to select endometrial cancer patient using “overexpression of dysregulated genes (DHODH) as the genetic markers for tailored therapeutic intervention using repurposed FDA approved drugs (Teriflunomide). Furthermore, it revealed a potential combination therapeutic strategy (Teriflunomide+ Olaparib) for the treatment of “DHODH overexpressing” endometrial cancer. Overall, this result provides preclinical data to establish future clinical therapeutic intervention to increase the efficacy of treatment response that improves overall survival of endometrial cancer patients.

**Abstract:**

Endometrial carcinoma (EC) is the most common gynecological malignancy in the United States. De novo pyrimidine synthesis pathways generate nucleotides that are required for DNA synthesis. Approximately 38% of human endometrial tumors present with an overexpression of human dihydroorotate dehydrogenase (DHODH). However, the role of DHODH in cancer cell DNA replication and its impact on modulating a treatment response is currently unknown. Here, we report that endometrial tumors with overexpression of DHODH are associated with a high mutation count and chromosomal instability. Furthermore, tumors with an overexpression of DHODH show significant co-occurrence with mutations in DNA replication polymerases, which result in a histologically high-grade endometrial tumor. An in vitro experiment demonstrated that the inhibition of DHODH in endometrial cancer cell lines significantly induced replication-associated DNA damage and hindered replication fork progression. Furthermore, endometrial cancer cells were sensitive to the DHODH inhibitor either alone or in combination with the Poly (ADP-ribose) polymerase 1 inhibitor. Our findings may have important clinical implications for utilizing DHODH as a potential target to enhance cytotoxicity in high-grade endometrial tumors.

## 1. Introduction

Endometrial carcinoma (EC) is the most common gynecological malignancy in the United States, with an estimated 61,880 new cases and 12,160 deaths in 2019 [1]. When diagnosed at an early stage without metastasis, the five-year survival rate was over 80% [2]. Over 50% of women with endometrial carcinoma present with early-stage EC, a low-risk disease, and are treated by surgery alone. However, women diagnosed with advanced or recurrent disease have a poor prognosis, with a 5-year survival rate of 17% [3] and poor responses to current therapies with an overall survival of 12 months [4]. In addition, chemoresistance remains problematic for at least 15% to 20% of patients [5,6]. Notably, treatment modalities in EC vary depending on the grade and the stage of the disease. To enhance treatment responses and improve patient survival in EC patients, new combination therapy strategies need to be discovered.

According to Lafita-Navarro et al. (2022), metabolic adaptations leading to an increased synthesis of nucleotides via de novo biosynthesis pathways are emerging as key alterations driving different cancer types [7]. Cancer cells utilize purine and pyrimidine synthesis pathways to produce and supply nucleotides and to satisfy the requirements of highly proliferative cells [8,9]. Both purines and pyrimidines can be synthesized via two alternative pathways as follows: the de novo pathways that metabolize ribose and amino acids in a series of enzymatic reactions and the salvage pathways that recycle nucleotides present in the cells or their environment through phosphorylation or dephosphorylation reactions [10]. De novo pyrimidine synthesis pathways have a six-step reaction and are performed using three critical enzymes: 1-Carbamoyl-Phosphate Synthetase 2, 2-Aspartate Transcarbamylase, and 3-Dihydroorotase (CAD); Dihydroorotate dehydrogenase (quinone) (DHODH); and 1-Orotate Phosphoribosyl Transferase and 2-Orotidine-5′-Decarboxylase/Uridine Monophosphate Synthetase (UMPS) [7]. The fourth step of this de novo pyrimidine synthesis in mammals is the conversion of dihydroorotate to orotate, which is catalyzed by DHODH [11].

Human DHODH is a flavin-dependent mitochondrial enzyme and is the only enzyme in the de novo pyrimidine synthesis pathway that localizes in the inner mitochondrial membrane, mediating the oxidation of dihydroorotate to orotate by reducing ubiquinone [12]. In the mitochondria, DHODH also interacts with respiratory complexes II and III [13]; thus, DHODH’s activity is necessary for the mitochondrial electron transport chain function in addition to the production of pyrimidines and maintaining the redox balance [10].

The up-regulation of DHODH expression in diverse cancer types is consistent with the results from previously published data [14], but the effect of such up-regulation on clinical outcomes has not yet been determined. Furthermore, the impact of DHODH on modulating DNA replication and its impact on genomic stability in endometrial tumors is currently unknown. In this study, using TCGA data, we show that DHODH overexpressing tumors harbor greater genomic instability, which is clearly evidenced by significantly higher levels of total mutation counts and the aneuploidy score. In addition, we found that tumors with DHODH overexpression harbor a significantly higher mutation count of replicative polymerases, including DNA polymerase epsilon (POLE) and DNA polymerase delta (POLD1). This is consistent with the findings that common somatic mutations in POLE and POLD1 genes are implicated in endometrial carcinogenesis [15]. In addition, the co-occurrence of DHODH overexpression with the POLE and POLD mutation is significantly higher in histological high-grade tumors (G3). Furthermore, our in vitro study shows that the de novo pyrimidine synthesis inhibitor, leflunomide, which is an FDA-approved drug for the treatment of rheumatoid arthritis, induces replication-associated DNA double-strand breaks (DSBs) and, in combination with the poly (ADP-ribose) polymerase (PARP) inhibitor (Olaparib), enhances cellular sensitivity in EC. Our results suggest that the DHODH status should routinely be assessed in EC patients, which may likely provide a novel therapeutic opportunity to target tumors that carry altered levels of DHODH expression. This work lays the foundation for future in-depth preclinical studies and for the development of novel therapeutic strategies to improve EC outcomes.

## 2. Material and Methods

### 2.1. Cell Lines and Drug Treatment

Endometrial carcinoma cell lines HEC1B and AN3CA were maintained in EMEM media supplemented with 10% Fetal Bovine Serum (FBS), 1% Penicillin/Streptomycin, and 1% L-Glutamine at 37 °C in a 5% CO_2_ environment. HEC1B (ATCC number: HTB-113; lot number 70012939) with species identification used the STR analysis of nine loci with a DNA profile (TH01: 6, 7; D5S818: 11, 13; D13S317: 11, 16; D7S820: 9, 11; D16S539: 11, 12; CSF1PO: 10, 12; Amelogenin: X; vWA: 18; TPOX: 8, 11) and AN3CA (ATCC number: HTB-111; lot number 70005249) with the STR analysis of nine loci DNA profiles (TH01: 9.3, 10; D5S818: 11, 14; D13S317: 12, 14; D7S820: 7, 7.1, 10; D16S539: 10, 14; CSF1PO: 12, 13, 14, 15; Amelogenin: X; vWA: 14, 20; TPOX: 8, 10). All the cell lines obtained from the American Type Culture Collection (Manassas, VA, USA) and STR profiling aligned with the Cellosaurus database, and a Certificate of Analysis is included in the Supplementary Materials (Figures S1 and S2). Cells were treated with teriflunomide at various concentrations for 24 h in culture. The cells were then washed with PBS and used for further analysis.

### 2.2. Clonogenic Survival Assay

A clonogenic survival assay was performed according to the previously published protocol, including minor modifications [16]. Both the control and leflunomide-treated cells were plated in six-well plates at a density of 500 cells per well and cultured to allow adherence overnight. Cells were then treated with leflunomide (0–5 mM) for 24 h. Following treatment, the cells were washed with PBS supplemented with fresh growth media and allowed to grow for an additional 10 days. The cells were then stained with 0.25% crystal violet in an 80% methanol solution for 30 min. The colonies were finally counted and scored using visual techniques.

### 2.3. Western Blotting

Cells were lysed using a radioimmunoprecipitation assay (RIPA) buffer supplemented with a protease inhibitor (Cat. 25765800, Sigma Aldrich, St. Louis, MO, USA) and a phosphatase inhibitor (Cat. P5726, Sigma Aldrich). After denaturing the samples at 95 °C for 5 min, 30 μg of the protein samples were separated using SDS-PAGE and transferred onto nitrocellulose membranes (Cat. 1620112, Bio-Rad, Hercules, CA, USA). Next, the membranes were blocked with 5% BSA for 1 h and then incubated with primary antibodies against γH_2_AX (Cat. 07-164, Millipore, Burlington, MA, USA), 53BP1 (Cat. ab170186, Abcam, Cambridge, MA, USA), the anti-DHODH antibody (Santa Cruz Biotechnology Cat# sc-166348) or α-tubulin (Cat. 2125S, Cell Signaling, Danvers, MA, USA) overnight. The following day, the membranes were washed with PBST (0.1% Tween) and incubated with an anti-mouse (Cat. NXA931, GE Healthcare, Chicago, IL, USA) or anti-rabbit (Cat. NA934V, GE Healthcare) secondary antibody for 2 h before developing with ECL substrates (Cat. 170506, Bio-Rad). The gel images were captured using the Chem-DocXRS image acquisition machine (Bio-Rad).

### 2.4. Immunofluorescence

In total, 50,000 cells were seeded in chamber slides and cultured for 24 h before siRNA and drug treatment (see above). The cells were then fixed with 3.7% paraformaldehyde (PFA) for 15 min and permeabilized with 0.5% Triton X-100 in PBS for 10 min. The cells were then blocked with 3% BSA for 1 h prior to incubation at 4 °C overnight with primary antibodies, including γH_2_AX (Cat. 07-164, Millipore) and 53BP1 (Santa Cruz Biotechnology Cat# sc-22760). On the following day, the cells were washed with PBS and incubated for 1 h with the secondary antibody, including the FITC-conjugated anti-mouse antibody (Cat. 715-095-150, Jackson ImmunoResearch Labs, West Grove, PA, USA) and TRITC-conjugated anti-rabbit antibody (Cat. 711-025-152, Jackson ImmunoResearch Labs). Finally, the cells were mounted with coverslips using mounting media containing a DAPI stain before visualization.

### 2.5. Comet Assays

A single-cell gel electrophoretic comet assay was performed under neutral conditions to detect DNA double-strand breaks. Cells were collected and rinsed twice with ice-cold PBS; 2 × 10^4^/mL cells were combined with 1% LMAgarose at 40 °C at a ratio of 1:3 (*v*/*v*) and immediately pipetted onto slides. For cellular lysis, the slides were immersed in a lysis solution (Trevigen, Gaithersburg, MD, USA, 4250-010-01) overnight at 4 °C followed by washing in the rinse buffer (90 mM Tris buffer, 90 mM boric acid, 2 mM Na_2_EDTA at pH 8.5) for 30 min two times. The slides were then subjected to electrophoresis at 20 V (0.6 V/cm) for 25 min and stained with SYBR green for 30 min. All images were taken with an FITC filter using a Zeiss fluorescence microscope (Zeiss, San Diego, CA, USA) and analyzed using the Open Comet Assay using the ImageJ (ver. 13.0.6) application as described previously [17].

### 2.6. DNA Fiber Analysis

For DNA replication analysis, the sequential labeling of DNA with IdU and CldU was performed based on previously described methods [18,19]. A sub-confluent, asynchronous population of HEc1B and AN3CA endometrial cancer cells was first labeled for 30 min with 25 μM IdU, washed with a medium three times, and treated with 100 μM treflunomide 1 for 6 h. The cells were then labeled for another 30 min with 250 μM CldU. After incubation, cells were washed and resuspended at a concentration of 7.5 × 10^5^ cells/mL. The number of cells lysed per slide ranged between 1500 and 5000 cells using a fiber lysis buffer (50 mM EDTA, 0.5% SDS, 200 mM Tris-HCl, pH = 7.5) for 2 min, and the slides were tilted at 20° for gravity flow. The control non-treated cells used were pulsed for 30 min with IdU, followed by 6 h with media only, before being pulsed with the CIdU label for 30 min, and the cells were harvested for the fiber assay. For immunofluorescence staining, the slides were fixed for 10 min with methanol/acetic acid (3:1) and air-dried. The slides were treated with 2.5 M HCl for 30 min, washed three times with one times PBS buffer, and then blocked with 3%BSA/PBS for 1 h. CldU was detected by incubating acid-treated fiber spreads with the rat anti-BrdU monoclonal antibody (Abcam), and IdU was detected using the mouse anti-BrdU monoclonal antibody (1:1000; Becton Dickinson, Franklin Lakes, NJ, USA) for 1 h at room temperature. This was followed by washing three times with 1× PBS and staining with a secondary antibody conjugated with sheep anti-mouse Cy3 and goat anti-rat Alexa flour 488 (Bioss Cat# bs-0346M-A488) for 1 h at room temperature. The slides were mounted with Vectashield mounting media and covered with coverslips. Images were acquired with 63× magnification using a Zeiss microscope and processed and analyzed using the ImageJ program. The lengths of red- (Cy3) or green (AF 488)-labeled patches were measured using the ImageJ software (National Institutes of Health; http://rsbweb.nih.gov/ij/, accessed on 6 September 2023, version 13.0.6) and arbitrary-length values were converted into micrometers using the scale bars created by the microscope. Fluorescence images were captured using a Zeiss microscope with a 63×/NA 1.4 oil immersion objective, and data analysis was carried out using the ImageJ software. The applied conversion factor we used was 1 μm = 2.59 kb [20].

### 2.7. Data Acquisition

TCGA datasets were pulled from cBioProtal (http://cbioportal.org, accessed on 6 September 2023) using uterine corpus endometrial carcinoma from the PanCancer Atlas dataset (*n* = 507). Since DHODH is the target of interest for this analysis, only individuals with valid RNA Seq V2 RSEM data for DHODH were included. Individuals were grouped as having either a low or high expression of DHODH; the *z*-scores of each individual were considered. Patients with POLE or POLD1 mutations and DHODH overexpression were included in this study. In addition, individuals with DHODH and neoplasm histological grading were included in the group analysis.

### 2.8. Statistical Analysis

Three independent experiments were performed for each group using the following: immunofluorescence, comet assay, cell proliferation, and cell survival assay. Data were statistically analyzed using Student’s *t*-test. Correlation analyses of DHODH expression associated with and without POLE, POLD1, and POLA1 mutations were evaluated using GraphPad Prism (GraphPad Software, Prism 10, Boston, MA, USA). The results were considered significant at *p* < 0.05.

## 3. Results

### 3.1. Targeting DHODH Induces DSBs in Endometrial Cancer

**Figure 1:**
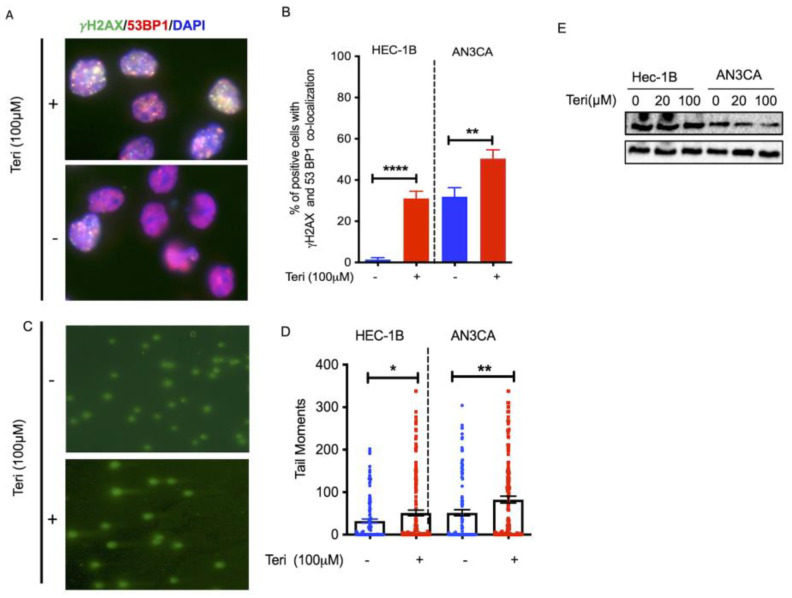
Aberrant DHODH induces genomic instability in endometrial cancer. (**A**) Representative image of γH2AX and 53BP1 in untreated versus teriflunomide (100 µM)-treated endometrial cancer cells for 24 h (γH2AX/53BP1 and DAPI); (**B**) Percentage of endometrial cancer cells with a γH2AX/53BP1 co-localization greater than 5 foci (HEC1B and AC3NA); (**C**) Representative image of neutral comet assay from untreated and treated endometrial cancer cells stained with SYBR green; (**D**) Percentage of quantification of comet tail moment in untreated versus teriflunomide-treated (24 h) endometrial cancer cells. Comet tail moments were analyzed using Open comet tools. (**E**) Western blot analysis of DHODH protein expression with teriflunomide treatment. All statistical analyses were performed using Student’s *t*-test, and statistical significances are represented as * *p* < 0.05, ** *p* < 0.01, **** *p* < 0.0001.

To determine whether de novo pyrimidine synthesis, specifically DHODH, was required to protect cells from DBSs, we used γH_2_AX and tumor protein 53 binding protein No. 1 (53BP1) with nuclear foci staining to monitor DSBs. Our data show that the number of endometrial cancer cell nuclei stained with γH_2_AX and co-localized with 53BP1 were significantly increased in DHODH inhibitor-treated cells versus untreated cells, HEC-1B: 31% versus 1.3% (** *p* < 0.01) and AN3CA: 52% versus 32% (*** *p* < 0.001), respectively (Figure 1A,B). We also performed a neutral comet assay to confirm DSBs in endometrial cancer cells before and after DHODH inhibitor treatment (Figure 1C), where we found a significant increase in the comet tail moment in treated versus untreated cells (Figure 1D; Mean ± SEM: HEC1B: 32 ± 5 versus 51 ± 7, * *p* < 0.05; ANC3A: 52 ± 7 versus 83 ± 8, ** *p* < 0.01). In addition, Western blot analysis showed that the treatment of endometrial cancer cells with teriflunomide did not affect the level of protein expression at 24 h treatment (Figure 1E and Supplementary Figure S3).

### 3.2. DHODH Is Required for DNA Replication Fork Progression

**Figure 2:**
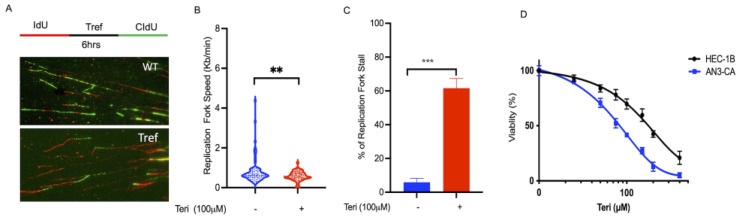
Inhibition of DHODH leads to replication stress and a delay in fork progression. (**A**) Representative image of DNA replication fiber from endometrial cancer cells. Note that the schematic representation of replication tracts in untreated versus treated cells was first labeled with IdU (25 µM) for 30 min (red line), then treated with teriflunomide (100 µM) for six hours (black line), followed by second labeling (green line) before being processed for DNA fiber analysis as described in the Materials and Methods section. (**B**) Estimated replication fork speed in endometrial cancer cells with and without teriflunomide treatment; (**C**) Estimation of cells with stalled replication forks in endometrial cancer after teriflunomide treatment; (**D**) Endometrial cancer cell (HEC-1B and AN3CA) viability measured using the MTT assay after treatment with teriflunomide for 24 h. All statistical analyses were performed using Student’s *t*-test and One-Way ANOVA. Statistical significances are represented as ** *p* < 0.01, *** *p* < 0.001.

We hypothesized that DHODH inhibition would exacerbate stress-related DSBs during replication in endometrial cells. In order to determine whether the DSBs were associated with actively replicating DNA, we performed the co-immunostaining of cells with antisera against CIdU and γH2AX as markers of replication stress [18,19]. To further determine whether DHODH is required for replication fork progression, we used DNA fiber labeling methods to measure the progression of the replication fork [18]. Next, to determine whether the DHODH is critical for cell cycle maintenance and nascent DNA strand (NDS) during replication stress, we used a single-molecule DNA fiber technique [21]. For each experimental condition, individual DNA fibers were uniformly stretched on glass coverslips and visualized via fluorescence microscopy using specific antibodies to detect IdU-labeled DNA (Figure 2A).

Replication tracts in endometrial cancer cells were first labeled with IdU (25 μM) for 30 min, then treated with teriflunomide for 6 h, followed by second labeling with CldU (250 μM) for 30 min (Figure 2A). Interestingly, the speed of the replication forks was significantly reduced to 0.5 kilobase (kb)/min in teriflunomide-treated versus untreated cells at 0.8 kb/min (Figure 2B; ** *p* < 0.01). Our data also show that endometrial cancer cells spontaneously exhibited 6% stalled forks, which increased significantly up to 61% after 6 h of teriflunomide treatment (Figure 2C; *** *p* < 0.001). Similarly, endometrial cancer cells showed a significant decrease in viability after teriflunomide treatment (Figure 2D; *** *p* < 0.001). Together, these data suggest that DHODH is required for replication fork progression both intrinsically and after pyrimidine synthesis inhibitor-associated replication stress.

### 3.3. Inhibition of DHODH Increases Sensitivity of Endometrial Cancer Cells to PARP Inhibitor

**Figure 3:**
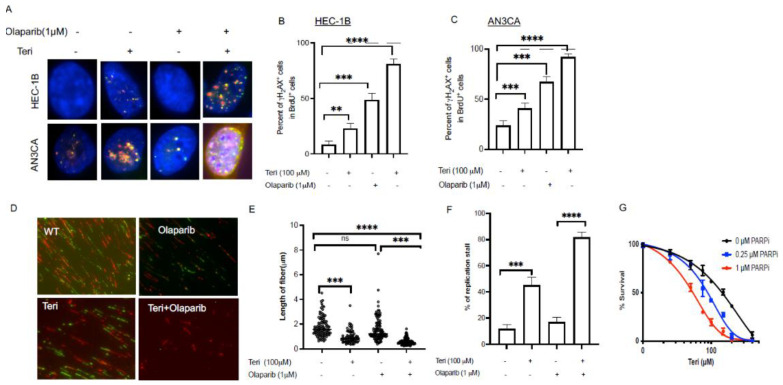
Targeting DHODH increases replication-associated DSBs and increases sensitivity to the PARP inhibitor. (**A**) Representative image of γH2AX (green foci)–and BrdU-positive cells (red foci) with and without teriflunomide, DNA stained with DAPI (blue); (**B**) Quantified percentage of cells with γH2AX and BrdU co-localization in HEC-1B and AN3CA (**C**); (**D**) Representative image of DNA fiber before treatment labelled with IdU (red) and after treatment with teriflunomide, the PARP inhibitor (Olaparib) and a combination (teriflunomide plus Olaparib) labeled with CIdU (green); (**E**) Estimated length of nascent DNA fibers with and without treatment; (**F**) Quantified replication forks stalled in endometrial cancer cells with and without treatment; (**G**) Viability were measured using MMT assays with two different concentrations of Olaparib (0.25 µM and 1 µM) in combination with different concentrations of the DHODH inhibitor (teriflunomide). Viability data were analyzed using a Two-Way ANOVA; multiple comparisons and statistical significance are designated as ** *p* < 0.01; *** *p* < 0.001 and **** *p* < 0.0001; ns represent not significant. Data were analyzed using Two-Way ANOVA and GraphPad Prism.

Previous studies suggest that PARP1 is associated with replication forks [22], and its inhibition leads to replication fork-associated DSBs [23]. Here, we tested whether treatment with a PARP1 inhibitor (Olaparib) in combination with a DHODH inhibitor (teriflunomide) synergized to increase DSBs in endometrial cancer cells. Our results indicate that when treated with a combination of these drugs, endometrial cancer cells show a significantly increased DSB formation compared with single agent-treated cells (Figure 3A–C; 23% versus 8% of HEC-1B ** *p* < 0.01; and 81% versus 43% of AN3CA cells, *** *p* < 0.001). We performed a DNA fiber assay to further evaluate the synergistic impact of teriflunomide and Olaparib on DNA replication (Figure 3D). For this experiment, endometrial cancer cells were pulsed with IdU for 30 min and then treated with Olaparib (1 μM) and teriflunomide (100 μM) for 6 h, followed by 30 min of CldU labeling. We then measured the length of DNA fibers that contained CldU tracts. Our data show that with teriflunomide treatment alone, the tract length of NDS was half that of untreated endometrial cancer cells (Figure 3E; Mean ± SEM: 0.9 μm ± 0.1 versus 1.8 μm ± 0.1, *** *p* < 0.001). Furthermore, the length of the NDS segments was significantly shorter in EC cells treated with a combination of teriflunomide and Olaparib (Figure 3E; Mean ± SEM: 0.6 μm ± 0.02; *** *p* < 0.001). Furthermore, the replication fork speed after treatment was quantified by dividing the length of each fluorescent track by the time of incubation with the halogenated CldU nucleotide, as shown in Figure 3D. Interestingly, we found that the combination treatment significantly increased the number of stalled forks by 82% after the six-hour treatment compared to the PARP inhibitor alone (17%) or DHODH inhibitor alone (45%) (Figure 3F; **** *p* < 0.001). To assess the cellular toxicity of a single or combined treatment of teriflunomide and Olaparib in endometrial cancer cells, we treated HEC1B endometrial cells with two different concentrations of Olaparib (0.25 μM and 1 μM) in combination with a range of teriflunomide concentrations (10 nm–100 μM) and evaluated the cell survival rates. Our data show that the percentage of cell survival was significantly decreased in EC cells treated with teriflunomide in combination with Olaparib treatment (Figure 3G).

### 3.4. DHODH Overexpression Associated with High Genomic Instability in Endometrial Cancer Patients

**Figure 4:**
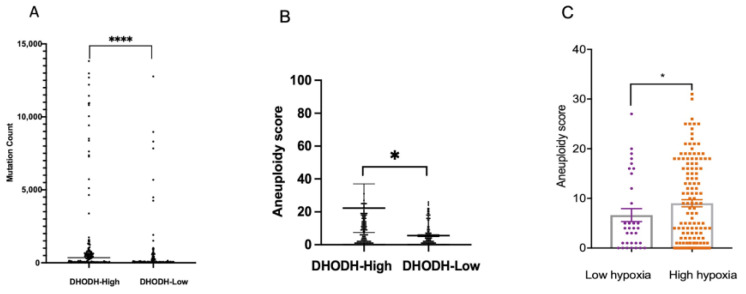
The overexpression of DHODH-associated genomic instability. (**A**) DHODH overexpression increases the mutation count in tumors with a high and low expression of DHODH; (**B**) Aneuploidy score of DHODH high expression versus low expression; (**C**) DHODH overexpressing tumors harbor significant genomic instability with high hypoxia versus low hypoxia. Statistical significances were analyzed using the Mann–Whitney U test * *p* < 0.05; **** *p* < 0.0001.

To assess the possible deregulation of DHODH in endometrial cancer patients due to genomic alteration, we first analyzed whether DHODH expression levels were associated with mutation load or aneuploidy in tumors of endometrial cancer patients obtained from The Cancer Genome Atlas (TCGA) database. We found that tumors derived from patients overexpressing DHODH accumulated mutations (Figure 4A) and a significant increase in the aneuploidy score, representing a fraction of the copy number variation (CNV) (Figure 4B). Moreover, the detected genomic instability, as measured using the aneuploidy score, significantly increased hypoxic tumor microenvironments (Figure 4C; *p* < 0.05).

### 3.5. DHODH Overexpression Associated with Mutation of DNA Replicative Polymerases in High-Grade Endometrial Tumors

**Figure 5:**
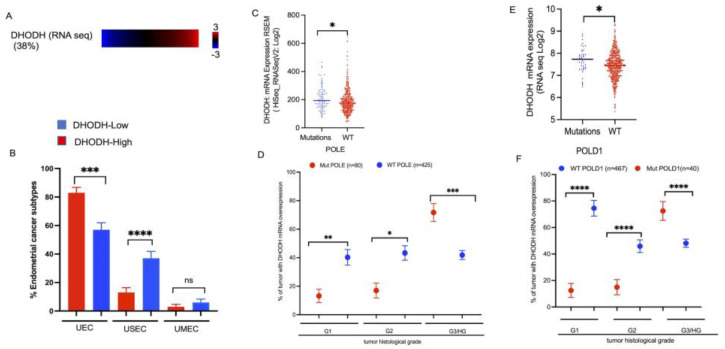
Overexpression of DHODH in different subtypes of endometrial cancer associated with mutated replicative polymerase (POLE, POLD1) in high-grade endometrial tumors. (**A**) The percent of patients with an overexpression of DHODH (TCGA dataset *n* = 507); (**B**) Overexpression of DHODH in different subtypes of endometrial cancer [uterine endometrial carcinoma (UEC), Uterine serous endometrial carcinoma/papillary serous endometrial carcinoma (USC); Uterine-mixed endometrial carcinoma (UMEC)]; (**C**) DHODH expression enhanced in tumors with POLE mutation; (**D**) Histological grade 3 endometrial tumors significantly increase in POLE-mutated groups versus non-mutated groups; (**E**) DHODH expression increases in POLD1-mutated tumors; (**F**) The histological grade 3 endometrial tumor is common in DHODH overexpressing and mutated POLD1 tumors. Data were analyzed using Two-Way ANOVA followed by Bonferroni’s multiple comparison test using GraphPad Prism; * *p* < 0.05, ** *p* < 0.01, *** *p* < 0.001, **** *p* < 0.0001, ns represent not significant.

To correlate our findings with human endometrial cancer, we quantified the expression data of DHODH in endometrial cancer using the TCGA database. We analyzed 507 patients from the Pan Cancer endometrial cancer dataset and observed that 38% of those patients overexpressed DHODH mRNA in tumor tissue samples relative to normal endometrial tissue (Figure 5A). Uterine endometrial carcinoma is a common cancer subtype (83%) harboring an overexpression of DHODH compared with uterine serous carcinoma/papillary serous carcinoma (13%, Figure 5B). By contrast, uterine serous carcinoma has a predominate papillary pattern subtype that harbors a low expression of DHODH (Figure 5B). In addition, endometrial tumors with DHODH overexpression also show an increase in mutated POLE and POLD1 genes (Figure 5C,D, * *p* < 0.5). Interestingly, 72% and 73% of endometrial tumors with DHODH overexpression co-occurrence with a POLE and POLD1 mutation are significantly associated with high-grade tumor stages, respectively (Figure 5E,F, ** *p* < 0.01, **** *p* < 0.0001). Our in silico data analysis suggests that the co-occurrence of DHODH overexpression and mutated DNA replicative polymerases can contribute toward unfavorable outcomes in high-grade tumor cases. In contrast, our data show that patients with DHODH overexpression and wild-type copies of POLE and POLD1 are significantly associated with low-grade (G1 and G2) endometrial tumors (Figure 5E,F, ** *p* < 0.01, **** *p* < 0.0001). Overall, our results indicate that the co-occurrence of DHODH overexpression with a DNA replicative polymerase (POLD and POLE) mutation status likely provides an opportunity for the selection of patients to receive combination therapy to enhance favorable outcomes.

## 4. Discussion

DHODH is a mitochondrial enzyme that catalyzes dihydroorotate oxidation to orotate [24]. DHODH plays a critical role in promoting cancer cell proliferation, which may be a highly sensitive target to inhibit nucleotide synthesis [25,26,27]. Emerging reports imply that DHODH is a potential target for cancer therapy [28]. In this study, we show that tumors with DHODH overexpression accumulate mutations and acquire high levels of chromosomal variations. Furthermore, endometrial cancer patients who exhibited DHODH overexpression had a higher risk of high-grade tumors. Our data are consistent with the observation of the presence of DHODH overexpression in other types of tumors [14,29,30]. One of the physiological factors that significantly alter cancer cell pyrimidine synthesis metabolism is hypoxia [31]. Our in silico result from the TCGA dataset demonstrates that DHODH overexpression in hypoxic tumor environments is associated with high levels of genomic instability. Interestingly, our in vitro data show that the inhibition of DHODH impairs DNA replication stability and promotes delayed DNA fork progression in endometrial cancer. Our data analysis shows that the co-occurrence of DHODH overexpression with mutations of replication polymerase genes (POLE and POLD1) is significantly increased in EC patients. Furthermore, the pathogenic mutations found in EC patient tumors compromise the exonuclease domain of POLE and the catalytic subunit of POLD1, which are involved in DNA replication, impairing cell proliferation by regulating the cell cycle, DNA synthesis, and cell differentiation [32,33].

Based on TCGA, molecular tumor profiling in the four distinct molecular subtypes of endometrial cancer is as follows: (1) hypermutation in the exonuclease domain of DNA polymerase-ε (POLEmut); (2) mismatch repair deficiency, which confers microsatellite instability (MMRd); (3) mutations in TP53; and (4) tumors with none of the aforementioned classifications (‘no specific molecular profile’ or ‘NSMP’) [15]. This molecular classification correlates with patient prognosis and can help improve the identification of early-stage patients who may benefit from adjuvant therapy. Previous studies have shown that endometrial cancer patients with a mutated POLE gene display high-risk features, such as tumors with a high-grade molecular subtype, and have exceptionally favorable survival outcomes [34]. Our data show that DHODH overexpression with a DNA replicative polymerase (POLE and POLD1) mutation is significantly associated with high-grade (Grade III) tumor status. Grade III EC is a common histological high-grade endometrial cancer in patients, which requires further characterization to improve clinical diagnosis, treatment, and prognosis approaches. The tumor molecular signature has a strong prognostic value in high-risk EC POLE-mutated patients’ better outcomes versus a worse outcome for p53 abnormal patients, with the MSI and NSMP groups having an intermediate outcome [35]. Even though previous studies have established a prognostic model for EC, they have not specifically targeted grade III high-risk patients [36,37,38,39,40,41]. Other studies explore the prognostic value of the endometrial cancer molecular classification for favorable and unfavorable prognoses, as well as the impact of different treatments [42,43]. In addition, the high level of DHODH expression in combination with other endometrial molecular signatures is likely used for patient stratification as well as the identification of subtypes of endometrial cancer. Furthermore, our study may provide a foundation to accurately classify grade III endometrial tumors based on DHODH overexpression associated with the POLE and POLD1 mutation, which may contribute to the better selection of patients for future therapeutic strategies.

The most common regimen of treatment for endometrial cancer patients is surgical resection followed by radiotherapy and chemotherapy [44,45,46]. Given the prevalence of DHODH overexpression in endometrial tumors, it may have a significant impact on developing targeted endometrial cancer treatment or influencing treatment response. Several attempts to use pyrimidine synthesis inhibitors as a treatment for cancer have been proposed [28,47,48]. Purines and pyrimidines are required for replicative DNA synthesis, and the metabolic pathways involved in nucleotide biosynthesis have historically been attractive targets for cancer drug development, such as methotrexate [49]. Accordingly, it is not surprising that multiple cancer types are vulnerable to inhibitors of de novo pyrimidine biosynthesis [50,51,52]. DHODH overexpression in tumors can lead to resistance to chemotherapy, so inhibitors of repair proteins may have the potential to sensitize selected types of cancer [48,53,54]. Our results demonstrate that the inhibition of DHODH in endometrial cancer cells leads to a decrease in cellular proliferation, which is in line with other works [14,55]. In addition, our data also strongly suggest that inhibiting DHODH alone and in combination with the PARP1 inhibitor promotes replication-associated genomic instability and significantly decreases the survival of endometrial cancer cells. This suggests that the inhibition of DHODH in combination with the PARP1 inhibitor (Olaparib) could become an alternative approach to treating endometrial patients. Our results also suggest that DHODH might be a potential prognosis marker for high-risk patients and/or an attractive therapeutic target for selected cancers. Overall, our findings suggest that there may be new therapeutic possibilities for endometrial cancer patients by blocking the de novo pyrimidine biosynthesis pathway.

## 5. Conclusions

Overall, the data shown in this work demonstrates that endometrial cancer patients with tumors exhibiting higher DHODH expression levels are significantly associated with histologic high-grade tumors. Our results could identify the overexpression of DHODH in selected types of cancer as a target or prognostic factor to better forecast potential outcomes and to determine which patients merit more aggressive therapeutic measures. In addition, the total number of somatic coding mutations in a tumor is emerging as a promising biomarker for immunotherapy responses in cancer patients. In particular, the mutational burden in DHODH overexpressing tumors likely provides a base for future researchers to test whether these cancer types respond to a class of cancer immunotherapy drugs. In the future, physiological or pathological circumstances of targeting DHODH in endometrial cancer have to be determined in vivo. This suggests that DHODH inhibitors should be further evaluated in vivo. Whether DHODH represents a viable target for endometrial cancer treatments in vivo needs to be determined.

## Data Availability

The original contributions presented in the study are included in the article. Further inquiries can be directed to the corresponding author.

## Supplementary Materials

The following supporting information can be downloaded at: https://www.mdpi.com/article/10.3390/cancers15245727/s1, Figure S1: DHODH overexpression associated DNA replicative polymerase expression. Figure S2: Distribution of DHODH overexpression and DNA replicative polymerase associated with tumor histological grade. Figure S3: Original Western blot of protein lysate from HEC-1B and AN3CA cell lines without and with teriflunomide treatment for 24 h and 48 h.
