# Supplementary material for: Inhibition of DHODH Enhances Replication-Associated Genomic Instability and Promotes Sensitivity in Endometrial Cancer
Source: Cancers (Basel). 2023 Dec 6;15(24):5727. doi: 10.3390/cancers15245727 (PMC10741824; doi:10.3390/cancers15245727)
Supplement: Supplementary file 1 [file cancers-15-05727-s001.zip › cancers-2635038-supplementary.pdf]

Data acquisition

TCGA Uterine corpus endometrial cancer data sets were pulled from cBioProtal (http://cbioportal.org). Since DHODH overexpression is the primary target of interest for this analysis, only individuals with valid RNA Seq V2 RSEM data for DHODH were included. The correlation of the change in DHODH expression and the mutation count in endometrial cancer and replicative polymerase (POLD1, POLD2, POLD3 and POLD4 , POLA1, POLA2, POLA3, POLA4 were analyzed using Graph Pad prism.

Supplement Figure S1. DHODH overexpression associated DNA replicative polymerase expression. A) Correlation analysis for DHODH versus the expression level POLD2, POLD3 , POLA1, POLA2, POLA3 and POLA3 . B)DHODH overexpression associated with POLD2, POLD3 , POLA1, POLA2, POLA3 and POLA4 mutation co-occurrence in human endometrial cancer (*n* = 506 samples) from TCGA Pan Cancer Atlas. *R* and *P* represent Pearson's correlation coefficients and 2-tailed *P* values.

Supplement Figure S2. Distribution of DHODH overexpression and DNA replicative polymerase associated with tumor histological grade. A) POLE mutation versus DHODH expression associated with tumor grade; B) POLD1 versus DHODH expression associated with tumor grade ; C) POLA1 versus DHODH expression associated with tumor grade.

Supplement Figure S3. The original western blot image of DHODH (43kDa) and alpha tubulin proteins (50kDa) from HEC-1B and AN3CA cell lines. Cells were treated with Teriflunomide for 24 hours and 48 hours. In addition, experimental control included (DMSO treated cells). Protein lysates were prepared from whole-cell extracts. Proteins were detected using a chemiluminescence system with a horseradish peroxidase-conjugated secondary antibody. The red marked area indicated the image taken (cropped)to the main text of the manuscript.

Supplement Figure S1

A

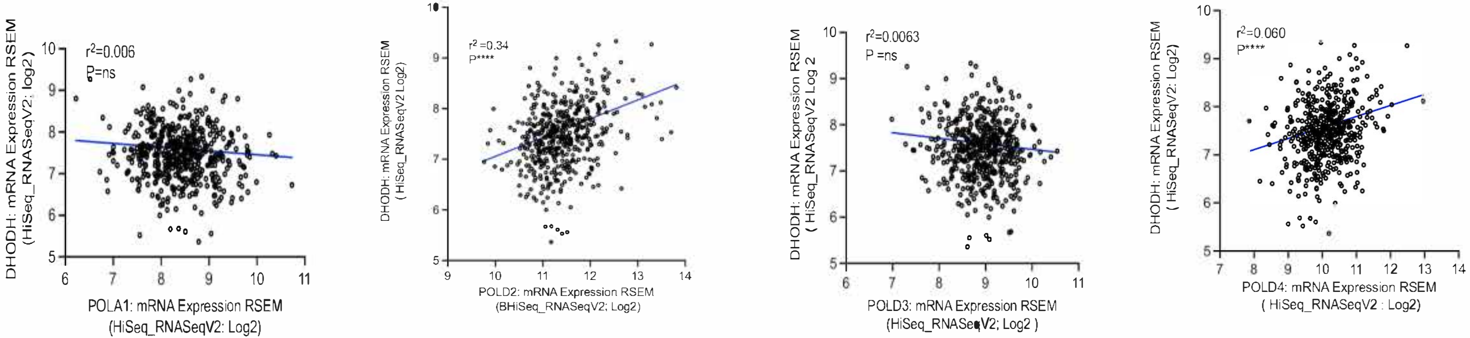

B

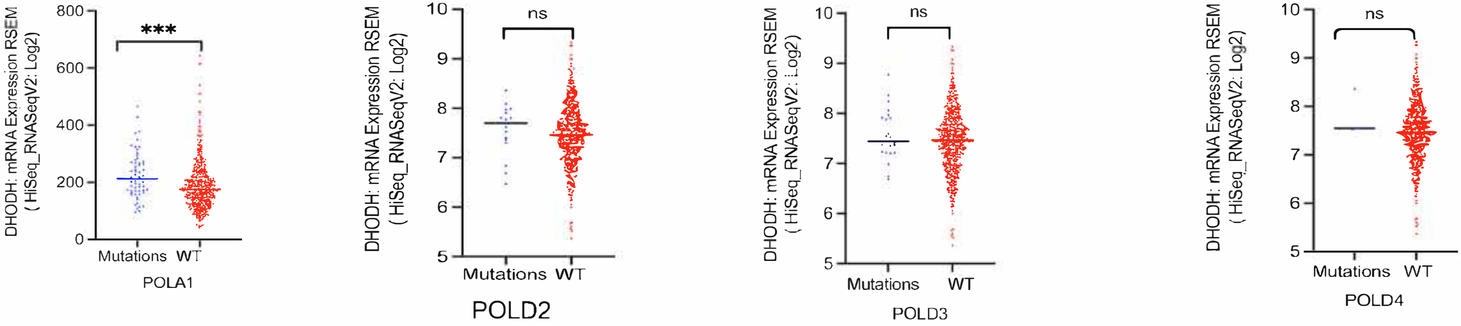

Supplement Figure S2

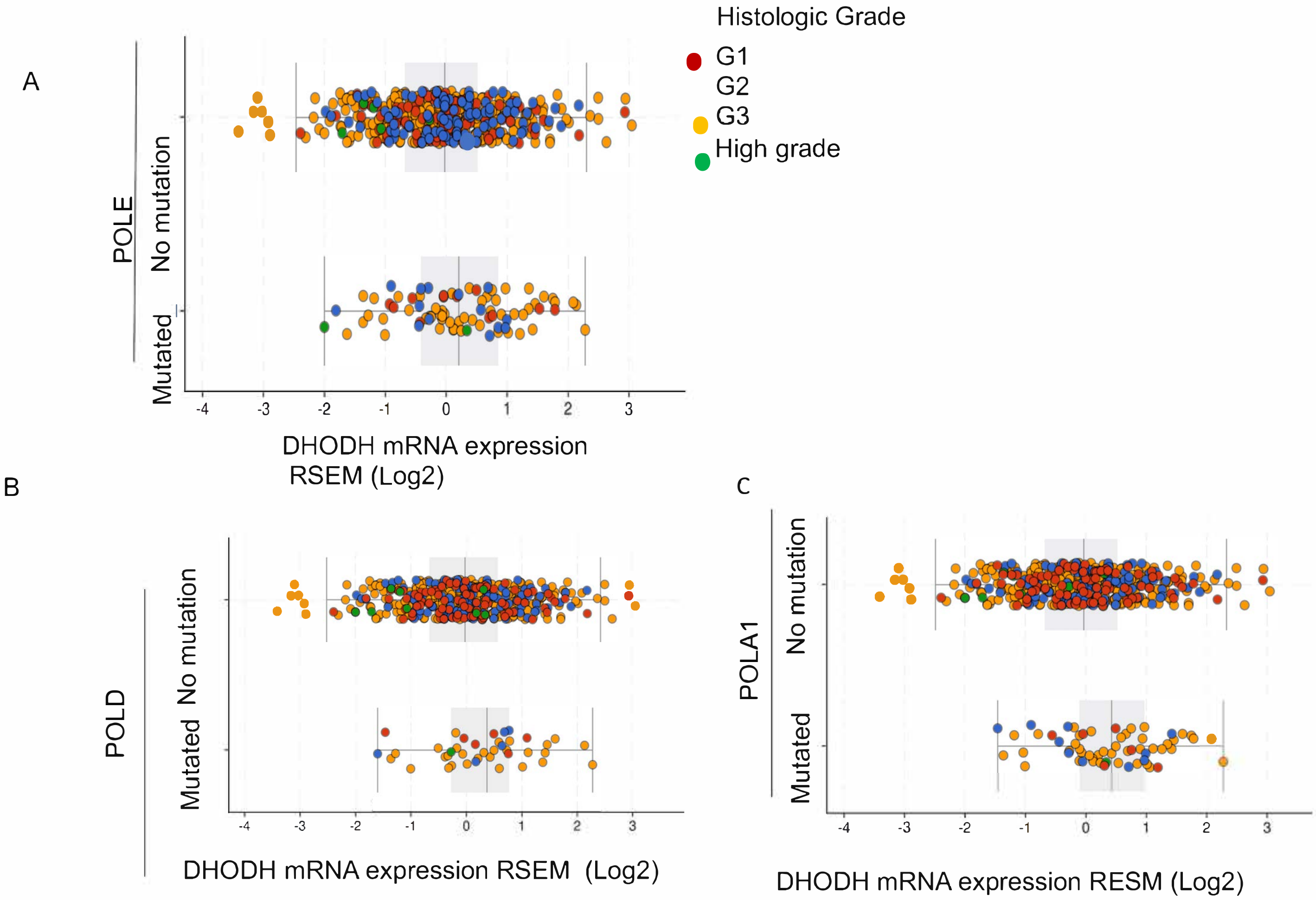

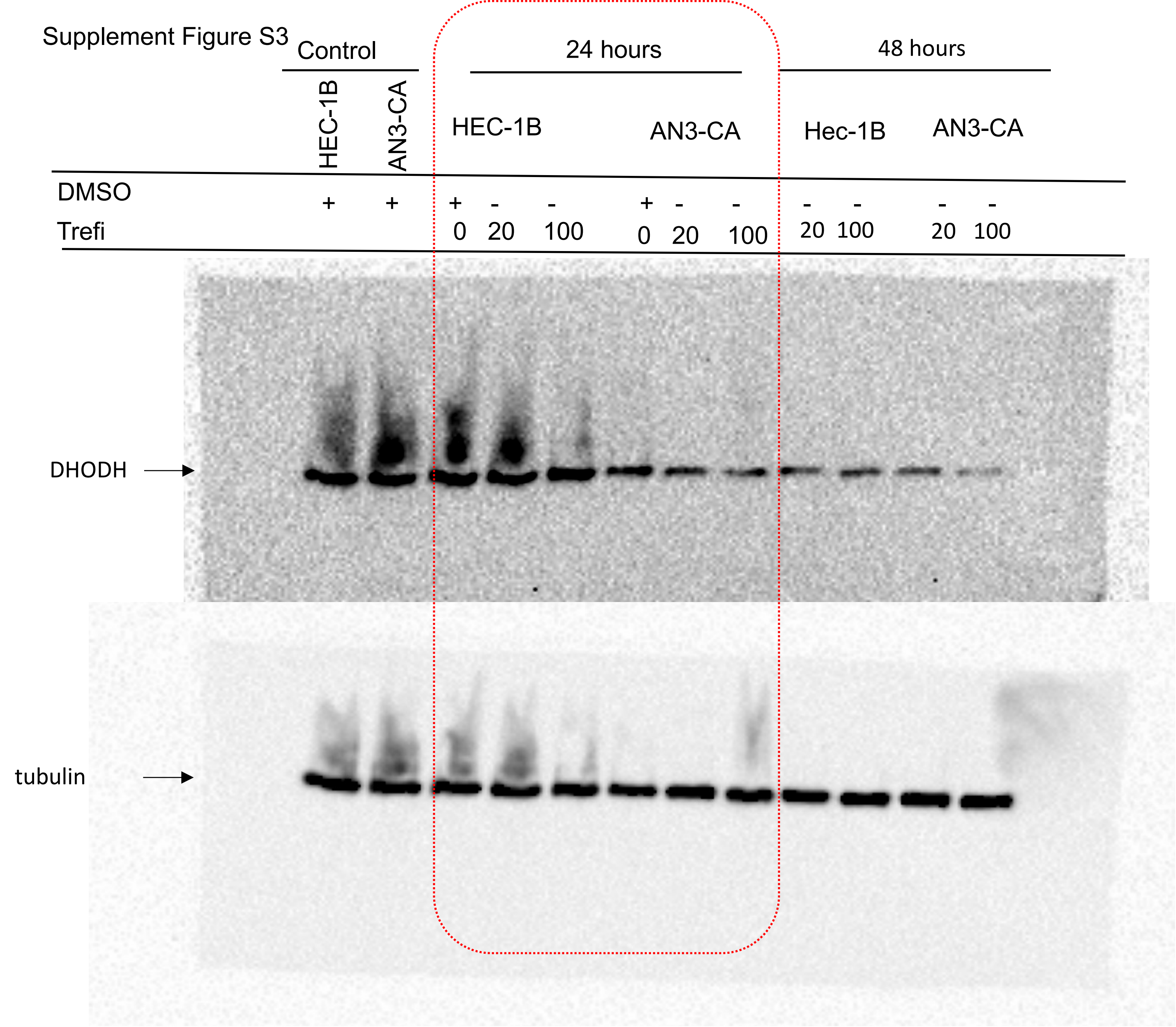

Supplement Figure S3
